# Editorial for the Special Issue on Microfluidic Brain-on-a-Chip

**DOI:** 10.3390/mi12091100

**Published:** 2021-09-13

**Authors:** Regina Luttge

**Affiliations:** Neuro-Nanoscale Engineering, Department of Mechanical Engineering/Microsystems and Institute of Complex Molecular Systems, Eindhoven Artificial Intelligence Systems Institute, Eindhoven University of Technology, P.O. Box 513, 5600 MB Eindhoven, The Netherlands; r.luttge@tue.nl; Tel.: +31-40-247-5235

A little longer than a decade of Organ-on-Chip (OoC) developments has passed [1]. The paper of Huh et al. marked the onset of this growing interest on reconstituting organ-level function on a chip by proposing a Lung-on-Chip [2]. Many more papers proceded from this landmark paper to discuss new in vitro models, such as summarized by Benam et al. [3], stressing the merging of tissue engineering and microfabrication as an asset in 2015. In toxicology and drug metabolism studies [4], many new miniaturized optical, chemical, and electrical sensors and analytical methods already appeared [5]. To elucidate design criteria and serve the unmet medical need in finding novel treatments for a great many devastating and poorly understood diseases, specifically brain diseases and disorders of the nervous systems, tissue engineers and translational medical experts, who previously worked on organoid technology from the stem cell route only, now fully embrace the prospects of implementing nano- and microfluidics [6,7].

First, fabrication methods must be created to harness the delicate interaction with neural cells sustaining them for weeks in culture. Subsequently, physical microenvironments must be devised to prove themselves for upscaling testing capacity in screening applications. Third, devices must be thoroughly designed and characterized for being fit for purpose demonstrating advances of human-specific in vitro based assays compared to 2D culture formats. Bae et al. summarized this evolving field of research from the perspective of enabling methods ranging from microfluidic chips to biomaterials for 3D culture and novel types of readouts [8]. The examples in their review confirmed the potential of these systems in tackling yet incurable diseases. Many disciplines must come together in the right place at the right time to showcase the working mechanisms of integrated biosensing in meaningful OoCs. To model parts of the human nervous system, including central nervous system or short “brain”, pharmaceutical workflows rely on abundantly available cell sources. The tissue engineering market now offers such abundant source thanks to human induced pluripotent stem cells even for such complex cells as neurons. Next, we need to connect them with multimodal readouts, which requires advances in chip packaging and standardization of interconnects for media and reagents exchange. Materials supporting such delicate cell culture frequently deviate from the associated methods of chip fabrication materials, like glass and silicon. Thus, miniaturized soft-matter integrated functional brain-on-chip devices form a new class of microsystems, not yet well-defined. The term “Brain-on-Chip” (BoC) can often be confused with the exquisite developments of organic electronics [9] and memristive-type of devices for bioinspired neuromorphic computing [10], which aims to advance computational power at low energy consumption. Seeing these developments, of course, one can imagine that biological brain models and artificial computational devices inspired by the biology of the brain with its electrophysiological communication paths performing complex operations in problem-solving applications in an incredible energy-efficient fashion, also merge someday. In silico spiking neural networks, emulating the working mechanism of biological neural networks is already investigated to perform such complex computational operations [11,12]. And early examples of merging such a biological and an artificial neural network are coming into the picture for motor neuroprosthetics control already for a few years [13]. Without going into detail of these attempts to advance Artificial Intelligence (AI)-driven solutions, silicon and similar semiconductor materials are long-standing materials for nano- and microelectronic chip integration serving an electronics consumer industry that is heavily invested in driving innovative markets based on Moore’s law [14]. This innovation is guided by a strong industry association and its International Technology Roadmap for Semiconductors (ITRS) [15]. Next, More-than-Moore thinking implements miniaturization and integration to diversify applications for the existing, but expensive, high-tech microfabrication methods in the semiconductor industry, when addressing low-volume applications in chip manufacturing reusing existing processing lines is key. Uniquely, diversification of the semiconductor industry processes into potentially low-cost OoCs can take directly advantage of flexible electronics microfabrication methods [16] that offer to inject the life-sciences and pharmaceutical industry with exquisitely new concepts, like microfluidic brain-on-chips.

Microfluidic brain-on-chips entail the research of in vitro mimicry and entanglement of brain tissue organization and function by applying micro- and nanofabricated features as addressed in this special issue. It is an even younger subdomain of the just forming research direction on OoCs [17]. “Organ” is a placeholder for implementing cell sources that may range from dissected mammalian tissues to cultures of stem cells essentially mimicking organ function in such models. “Chip”, on the other hand, stands for integrated technologies by means of which we yield an understanding of all types of processes involved during interrogation of the microtissues in culture, including neural tissues. On-chip solutions to culture models must keep in mind the quest for cost-effective implementation of such techniques in the pharmaceutical and biomedical research workflow. Note, this new OoC technology is not just about applying a chip as a miniaturized replacement of a passive culture flask, like in microwell plate, or the mimicry of the properties of blood flow for its most rudimentary function. When we refresh nutrients and exchange cell waste products from the culture to the outside world thanks to microfluidics, we also aim to emulate physiological processes distinguishing between healthy or diseased states of tissues relevant for the clinical translation of these observations. OoC culture conditions should upconvert systems to reach a higher predictive value and accuracy, consequently serving the patient. Soft matter cell (bio)patterning introduces entirely new demands to chip fabrication technologists [18]. In addition, OoCs need a high level of modularity and establishment of an international technology roadmap is yet far from achieved. Such challenges in standardized upscaling of featured shaping the physical environment of cells in culture in miniaturized systems is barely reflected in the previously published articles, except for a few [19]. An exciting cross-multidisciplinary young research field, which is mostly concerned with showcases and consolidation, needs to lower first the many barriers in communication among the different scientific disciplines. The current special issue contributes to these efforts in lowering barriers for microfluidic brain-on-chip. The collected publications discuss underlying design requirements, constraints, and preferences to fabricate, vascularize, and manipulate biohybrid constructs dedicated to elucidating healthy and diseased brain functions or emulating them for advanced novel therapeutic concepts. This new class of microsystems will serve pharmaceutical as well as nervous system health-tech approaches of the future. Such future technology perspective on AI-based algorithms, which run on implantable biohybrid integrated electronic systems to treat neurodegenerative diseases, are envisaged here, in this special issue, already in the review on electrophysiology read-out tools for brain-on-chip biotechnology by Forro et al. [20].

Hierarchical organization of communication processes in these systems stand at the basis of actions that are central to cognition, behavior, and overall human health and (mental) well-being. Moreover, the design of microfluidic brain-on-chips using cells from human origin provides us with a technology for discovering and testing novel therapeutic interventions in a safe, ethically sound, and highly representative manner. The concept of microfluidic brain-on-chips extends to the entire nervous system as a cornerstone in conquering brain diseases and stimulating systems thinking of human health.

In more detail, microchannels and reservoirs can be fully or partially filled with hydrogels as highly porous scaffolding materials in parallel with flow-through concepts similar to the workings of the microvasculature in tissues, which helps circumvent diffusion limitations. Microfluidic brain-on-chips are also great to control mechanically steered cell interactions in neural networks cultured from dissociated cells into the third dimension. For example, rat cortical cells and neuroblastoma cells (SH-SY5Y) [21] have already been investigated by us simply by adding hydrogels into the on-chip culture reservoir. To this end, we presented here our additional article on the design and testing of a straightforward concept to evaluate whether 2D seeded cells transition into 3D, when cultured within a hard–soft interface adding a hydrogel atop of the seeded cells [22]. This concept allows us to assess if neurons are susceptible in surviving in these materials and how they change their morphology and organization upon the change in mechanical cue, i.e., offering a far lower Young’s modulus compared to the glass and multitude of 3D spreading anchoring points for cell interactions within the porous 3D matrix of the hydrogel. Easy-to-build assessment platforms with volume-confined reservoirs in screening applications for the validation and optimization of new scaffold materials and their properties for differentiation, migration, or barrier function, etcetera, are urgently needed and could be directly combined with encircling microfluidic channels, such as in our MEMS-based microbioreactor concept already demonstrated [23].

Furthermore, complex tissues, like brain, have been harvested ex vivo by dissecting tissues, either from biopsies, surgeries, or post-mortem. And, given limited access to human tissues, many sophisticated mouse models have been developed. Whole tissue slices preserve most of the 3D structure and matrix components. Dissociated cells, however, have completely lost organization and there are limited mechanical cues, when seeding cells in 2D on surfaces. Although culture surfaces are often coated with cell membrane mimicking adhesion molecules to make the physically engineering environment bio-friendly and trigger the cells from being in a pre-defined state of differentiation of glia or neuron in solution back into the state of forming neurite outgrowths upon adhesion. Possibly, these cells also form tripartite synapses [24] but they will nonetheless be connected in oversimplified and biologically less meaningful neural circuit networks compared to the in vivo state of tissue that they have been harvested from. This fact is side-stepped by putting ex vivo brain slices directly in petri dishes, multi-well plates, or also in microfluidically supported microelectrode array (MEA) plate formats. Since human brain tissue slices are an extremely rare source of cells to use experimentally these are not suitable for up-scaling to industrial formats in pharmaceutical screening studies. Hence, 2D neural cell culture formats from dissociated animal cells or human cell lines of tumor origin are the norm in life-sciences and the pharmaceutical industry. Either way, there is a long and sophisticated learning curve involved in running such experiments excessively prone to error. Automation of these standard 2D techniques and the simplicity of using epi-fluorescent microscopy based on the multiplying well-plate format in optically highly defined 2D cultures adds to the rapid screening of cells when applying fluorescent labels. Hence, passive 2D culture systems will remain a gold standard in drug target, metabolic, and tox studies for some time.

Alternative options for the implementation of efficient workflows and reduction of labor intensity for 3D cultured brain tissue, i.e., brain organoids, can be handled by robotic liquid handling and automated workstations for microsectioning of such tissues. Additionally, robotic liquid handling during initial cell seeding as well as postprocessing of such cultures, e.g., preparation of stainings prior to analysis by optical readout assays, also lead this direction of brain model technology developments. Despite these advances, such automated systems are expensive to install in research labs and they will not solve the issue of low predictive value for translational medicine in treating neurodegenerative diseases directly. Obviously, self-organized 3D stem cell-derived brain organoids promise a lot. And, with their way of culturing them in, for example, spinning bioreactor flasks or round-bottom culture dishes, these self-organized neural tissues became popular as mini-brains [25]. Unfortunately, these systems still provide very low efficiency due to the complexity in culture conditions and are delicate to handle throughout stem cell expansion and differentiation states until matured neurons and their networks can be studied in meaningful assays [26]. As superb as brain organoids [27,28] are they have not proven higher predictive value than 2D cultures and experimental preparations and pipetting for media change and staining in a low-throughput culture regime come at significant labor strength and clearly limit this approach. Next to missing regulations for advanced in vitro models, research labs lack investments and companies are not yet significantly funding research activities directly due to the absence of a sufficient proof of concept. At the verge of a large portfolio of possible applications, we fundamentally do not yet understand enough of this emerging brain model technology to underpin main hallmarks of the mechanisms worthwhile to optimize for better prediction. Without such confirmation of what makes a better model, we do not know the importance of parameters that we should invest in either. Upscaling the model’s platform fabrication to higher throughput formats that are urgently needed in the industry is basically waiting for validated showcases in academia. Confirmation of such hallmarks would be a starting point for prosperous technology road-mapping. Above all, we need systems and data harvesting techniques that provide us information of continuous variation of the cell dynamics. Raaijmakers et al. [29] provide us, in this special issue, insights in software-driven labor reduction in live-cell calcium imaging applications for brain-on-chips. Additionally, the contribution by Forro et al. [20] discusses the most recent developments of brain model read-outs utilizing electrical means.

Evaluation of the demands for extending cultures in physically engineered 3D microenvironments will be the following step in development. Planar MEAs are well-known in the art of neuro-electrophysiology to perform dynamic studies of neural networks and add value as a simple-to-automate read-out in microfluidic brain-on-chips culture concepts, such as published by us recently [23]. Here, we also contributed by demonstrating improvements on the polymer microfabrication of microsieves [30]. These devices serve the idea to transform classical planar MEA systems into 3D read-outs but with the same ease as 2D cultures [31]. In this way, physically engineered microenvironments, i.e., brain-on-chips, can take advantage of many recent advances of handling single cells by microfluidics and integrated single cell targeting analysis techniques integrated in a chip format. Existing nano- and microfluidic devices for single-cell analysis over bulk-cell analysis have many thought-provoking advantages over conventional techniques in cell culture. Next, implementation of microfluidics utilized as vasculature will most likely support differentiation and maturation of stem cell-derived human tissue cultures in 3D providing, for example, low-shear physical microenvironments. Microflow control in the range of micro to pico-liters with diffusion-limited transport of compounds will offer design features in conditioning the culture settings, i.e., biological cell patterning by kindling cell expression processes by time-targeted growth factor addition and local dosing for biomedical applications with tailored experimental requirements. Cameron et al. [32] reviewed new functionalities by design considerations for brain-on-chips models that offer additional advantage of microfluidics devices and can pave new avenues in this novel research field of organ-on-chips applications.

Needless to say, these investigations will lead to novel therapeutics and diagnostics approaches as it is also clearly laid out by Ustun et al. [33] in their contribution to brain tumor modeling on chips.

In conclusion, devastated diseases of the brain and the vast lack of understanding the complex nature of the human nervous system and its functions has been a long-standing incentive to develop tools for whole brain imaging and in vitro neurophysiological methods. To elucidate neural cell behavior and correlated genomic and proteomic expression in such complex tissue across multiple length scale can be realized by microfluidic brain-on-chips solutions. This special issue not only presents an excellent introduction into the field of microfluidic brain-on-chips by means of the contributed reviews and perspective papers, but also it discusses novel approaches of my own research group and colleagues in the Mechanical Engineering and the Electrical Engineering departments at Eindhoven University of Technology, serving as examples of the multidisciplinary landscape of novel microfluidic brain-on-chips technology.

## Data Availability

Not applicable.
